# Diet and Exercise: a Match Made in Bone

**DOI:** 10.1007/s11914-017-0406-8

**Published:** 2017-11-02

**Authors:** Hubertine M.E. Willems, Ellen G.H.M. van den Heuvel, Ruud J.W. Schoemaker, Jenneke Klein-Nulend, Astrid D. Bakker

**Affiliations:** 1 Department of Preventive Dentistry, Academic Center for Dentistry Amsterdam, Vrije Universiteit Amsterdam and University of Amsterdam, Amsterdam, The Netherlands; 2 FrieslandCampina, Amersfoort, The Netherlands; 3 Department of Oral Cell Biology, Academic Center for Dentistry Amsterdam, Vrije Universiteit Amsterdam and University of Amsterdam, Amsterdam Movement Sciences, Gustav Mahlerlaan 3004, 1081 LA Amsterdam, The Netherlands

**Keywords:** Dietary components, Nutrition, Diet, Osteocytes, Bone health, Physical activity

## Abstract

**Purpose of Review:**

Multiple dietary components have the potential to positively affect bone mineral density in early life and reduce loss of bone mass with aging. In addition, regular weight-bearing physical activity has a strong positive effect on bone through activation of osteocyte signaling. We will explore possible synergistic effects of dietary components and mechanical stimuli for bone health by identifying dietary components that have the potential to alter the response of osteocytes to mechanical loading.

**Recent Findings:**

Several (sub)cellular aspects of osteocytes determine their signaling towards osteoblasts and osteoclasts in response to mechanical stimuli, such as the osteocyte cytoskeleton, estrogen receptor α, the vitamin D receptor, and the architecture of the lacunocanalicular system. Potential modulators of these features include 1,25-dihydroxy vitamin D_3_, several forms of vitamin K, and the phytoestrogen genistein.

**Summary:**

Multiple dietary components potentially affect osteocyte function and therefore may have a synergistic effect on bone health when combined with a regime of physical activity.

## Introduction

The incidence of osteoporosis-related fractures is expected to rise substantially over the coming decades [1]. It has been estimated that the annual number of fractures in the European Union will rise from 3.5 million in 2010 to 4.5 million in 2025, with a corresponding increase in costs, which was estimated at €37 billion in 2010, including pharmacological intervention [2]. More importantly, osteoporosis-related fractures often lead to a diminished quality of life, disability, discomfort, and even death [2].

One safe way to prevent fractures is to positively affect bone mass and strength through mechanical stimuli. Lack of physical activity, e.g., as is seen in bedridden patients, results in a lack of mechanical stimuli and a rapid and substantial loss of bone mass [3]. High impact physical activity, on the other hand, has an anabolic effect on bone mineral content (BMC) and bone mineral density (BMD) [4]. The BMC and BMD have been shown to increase up to 20% in the loaded bone regions in athletes [5, 6]. In general, high magnitude mechanical loads as well as loads applied at high frequency are known to increase bone mass, while low impact activities have less effect on bone, even when applied for a longer duration [7, 8••]. Unfortunately, exercise at a high intensity might be difficult for those elderly at risk of fractures, and the key may lie at building a sufficient “reserve” of bone mass at a young age. Indeed, bone mass at the age of 70 largely depends on peak bone mass reached before the age of 30 [9]. During growth, not only high impact activity but also low impact activity contributes to skeletal health [10–12]. High impact physical activity in childhood, especially when initiated before puberty, results in increased bone width and increased bone mineral content in girls and adolescent females [13]. It is much debated whether the efficiency of physical stimuli for increasing bone strength diminishes with age, especially after the menopause. Exercise probably does benefit bone health in adults since bone resorption and bone turnover markers are reduced by 1 month of moderate intensity exercise, i.e., four times per week and 30 min per day, in both pre- and postmenopausal women [14]. This suggests that mechanical loading of bones through exercise benefits bone mass at a young age as well as at a later age.

More than a century ago, Roux (1850–1924) proposed a concept in which mechanical loading of bone leads to the cell-mediated adaptation of bone structure, resulting in an optimized load-bearing capacity of bone [15]. It then took several decades until the groundbreaking experiments by the groups of Nijweide and Burger in the Netherlands identified osteocytes as the chief mechanosensing cells in bone [16, 17]. Definite proof that osteocytes sense mechanical signals and respond by directing osteoclast activity came from animal experiments performed by Tatsumi et al., demonstrating a lack of bone loss in unloaded hind limbs of mice missing the majority of their osteocytes [18]. Activation of osteocyte signaling through exercise thus provides a way to prevent osteoporosis by maintaining, or even enhancing, bone mass.

Besides daily physical activity, a healthy diet is among the most commonly advocated lifestyle measures to improve (skeletal) health [19]. Recently, the National Osteoporosis Foundation wrote a position statement on peak bone mass development. The best evidence (grade A) is available for positive effects of calcium intake and physical activity, especially during the late childhood and peripubertal years—a critical period for bone accretion [19]. “European guidance for the diagnosis and management of osteoporosis in postmenopausal women” recommends a daily intake of at least 1000 mg/day for calcium, 800 IU/day for vitamin D, and 1 g/kg body weight of protein for all women aged over 50 years [20]. Persons need to consume an overall healthy diet, like increasing plant-based foods or dairy foods, to meet their nutrient requirements [21].

**Fig. 1:**
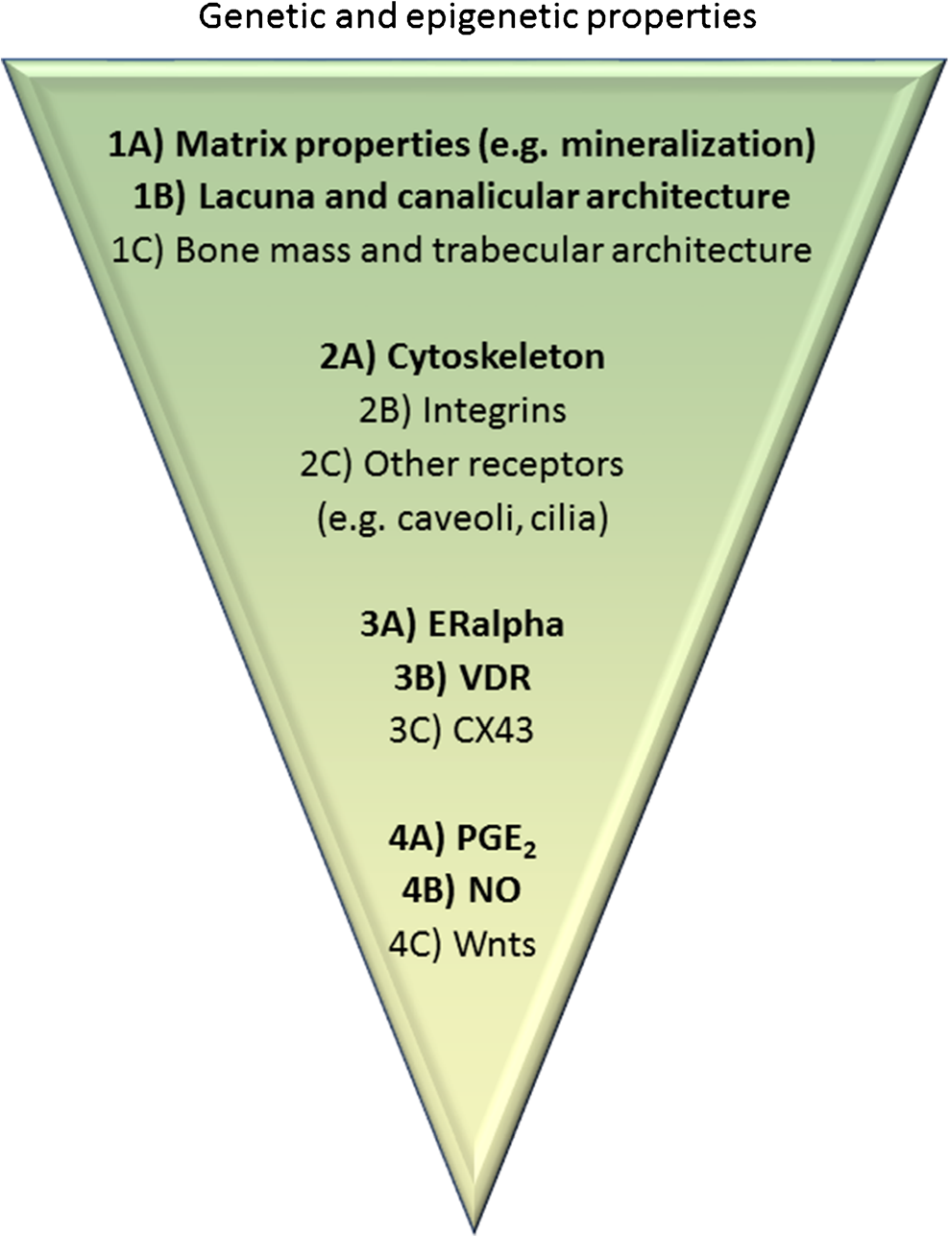
Mechanotransduction. Physical mechanical loads elicit a biological response through the process of mechanotransduction. For bone, this process can be broken down in the following steps: (1) Transmission of the bulk mechanical stimulus to the osteocyte, (2) Sensing of the mechanical stimulus by osteocytes and transduction into a chemical response, (3) Modulation of intracellular signaling, (4) Production of signaling molecules. The last step leads to an altered osteoclast and osteoblast recruitment and activity, and an alteration in bone mass and structure. Changes on each of these levels of mechanotransduction, for example, by affecting the cellular structures described in the figure, will potentially affect the efficacy of mechanical stimuli for modulating bone mass and structure, and thereby the ability of bone to withstand fracture. Governing all steps of the process of mechanotransduction is the genetic make-up of the osteocytes

The aim of this review is to identify potential interactions between dietary components and mechanical stimuli with respect to their effect on osteocytes and bone health. To this end, we defined cellular aspects of osteocytes that determine their signaling towards osteoblasts and osteoclasts in response to mechanical stimuli (Fig. 1) and by describing a number of dietary components with the ability to affect these cellular structures, thereby potentially enhancing the response of osteocytes to mechanical loading.

## The Combined Effect of Nutrition and Mechanical Loading on Bone In Vivo

Since mechanical loading is beneficial for bone mass and nutrition affects bone mass, the question then rises what is known about the combination of exercise and diet with respect to bone mass in animals and humans. It is difficult to give a clear answer, as positive effects, negative effects, and a lack of effects have been reported regarding the effect of exercise and dietary changes on bone mass. To provide a few examples, in adult male rats, bone morphology and strength are non-significantly affected by 30% food restriction combined with voluntary exercise training after a 13-week experimental period [22]. In young female rats, on the other hand, food restriction in combination with voluntary exercise significantly reduced bone strength, bone mineral density, and calcium absorption compared to an exercise group. This deficit could be partially rescued by calcium supplementation, suggested that calcium supplementation has a positive effect on bone strength in combination with exercise [23•]. On the other hand, dietary methionine restriction (MR) and endurance exercise down-regulate bone and energy metabolic indices, bone size, and extrinsic bone strength in growing rats, which appears to be part of an adaptive response to changes in energy metabolism in the entire organism. On a positive note, there is the possibility that MR and endurance exercise reduce bone aging by slowing bone turnover and enhancing energy metabolism [24].

In humans, a *high* intake of dairy, calcium, and protein during diet- and exercise-induced weight loss in premenopausal overweight and obese women favorably affects bone health biomarkers [25]. Inversely, *low* calcium intake diminishes the increased bone mass resulting from exercise in prepubertal girls [26]. One study conducted on the effect of diet and physical activity in healthy subjects (age 14–18 yrs.) shows that the main dietary variables related to bone mass are energy intake, calcium, vitamin D, and servings of dairy products, in combination with vigorous (jumping) physical activity [27]. Another study in well-trained female cyclists showed that a calcium-rich pre-exercise breakfast meal containing ~ 1350 mg of calcium consumed as compared to no calcium ~ 90 min before a prolonged and high intensity bout of stationary cycling attenuates the exercise-induced rise in markers of bone resorption [28]. A 2-year study showed that exercise was effective in reducing fall-related injuries among community-dwelling older women at a moderate cost. Vitamin D supplementation had marginal additional benefit [29]. An observational study in young adult men indicated that habits of consuming breakfast and exercising at least 10 h per week during high school were linked with significantly higher L2–4 and femoral neck BMDs [30]. In osteoporotic sedentary women, an intervention with soy isolate protein or soy in combination with progressive resistance exercises four times/week for 12 weeks significantly stimulates bone and muscle strength gains. Interestingly, the improvements are more pronounced in the soy-and-exercise group [31]. The combination of improving nutrition (adequate energy and vitamin D) and resistance exercise during spaceflight attenuates the expected BMD deficits previously observed after 4- to 6-month missions [32], showing that a proper combination of nutrition and exercise serves to maintain bone mass, even under extreme conditions.

The major drawbacks of studies on bone in vivo in animals and humans are the associated high costs and the long-term duration of the experiments. Moreover, the added benefit of a combination of physical activity and optimal dietary component status seems rather difficult to prove. However, it remains a tantalizing idea that osteoporosis can be prevented by providing the optimal combination of exercise and nutrition. A targeted approach to solving the question whether diet and exercise can synergistically benefit bone mass and strength is to start to answer the question whether it is possible to identify dietary components that enhance the response of osteocytes to mechanical stimuli. In multiple cases, the effects of dietary components on osteocytes have not been investigated, and effects on osteoblasts are described instead. Osteocytes are terminally differentiated osteoblasts, but care has to be taken when extrapolating results obtained with osteoblasts towards osteocytes, because they also have distinct differences in morphology and function as outlined below.

### Degree of Differentiation

Osteocytes are derived from osteoblasts, and osteoblasts and osteoblastic cell lines show mechanosensitivity [33••]. When osteoblasts differentiate into osteocytes, they become more sensitive to mechanical loading [33••]. Thus, by stimulating the differentiation of osteoblasts into osteocytes, it may be possible to enhance the sensitivity of bone tissue to mechanical stimuli, and therefore dietary components that are able to stimulate osteoblast differentiation might ultimately lead to changes in bone mass. Several of such components exist. The component fluoride is well known for its effect on osteoblast differentiation. It increases osteoblast proliferation and differentiation in a rat osteosarcoma cell line [34]. Another component that enhances osteoblast differentiation is lactoferrin, a pleiotropic factor and well-known dairy ingredient [35]. Lactoferrin stimulates both osteoblast proliferation and differentiation into osteocytes [36]. Other components that stimulate osteoblast differentiation are phytoestrogens, such as genistein, daidzein, diarylheptanoid and 8-prenylnaringenin [37, 38], and therefore it is possible that they affect the mechanoresponse of cells from the osteoblast lineage as well. Vitamin K2 inhibits miR 133a expression, which is accompanied by enhanced osteogenic differentiation of mesenchymal stem cells, but whether vitamin K also enhances the differentiation of osteoblasts towards osteocytes remains to be determined [39]. Additional components known to enhance osteoblast differentiation are strontium, isoflavones, and whey protein [40–42]. Whether any of these dietary components actually leads to an increase in the anabolic response of bone tissue—as a whole—to mechanical loading remains to be investigated. The increase in mechanosensitivity of terminally differentiated osteoblasts is likely related to the rigorous changes in the cytoskeleton associated with the transition of an osteoblast into an osteocyte [43]. How osteocyte mechanosensitivity and the cytoskeleton are related, and how this could be affected by dietary components, is elucidated next.

### Cytoskeleton

Lately, the view of the cytoskeleton as a structure responsive to external physical and chemical stimuli has become prominent [44]. The cytoskeleton is involved in mechanosensing and is a key determinant of the material properties of the cell. Osteocyte morphology, determined by the cytoskeleton, is correlated to the magnitude of the response to mechanical stimuli, whereby round osteocytes seem to have a higher response to mechanical stimuli than more spread, adherent osteocytes [45]. The shape of cytoskeletal structures depends on the dynamics of actin fibers, intermediate filaments, and microtubules [46, 47]. Actin fibers are most abundant in eukaryotic cells and form a framework that supports and shapes the plasma membrane. Intermediate filaments are relatively under investigated in mesenchymal cells, although they play an important role in the resistance of cells against shear forces. Microtubules exhibit structural and functional polarity and are important components of primary cilia [48], a mechano-sensitive structure that is present on osteocytes [49].

When thinking of cytoskeleton-affecting dietary components, a few come to mind. First, fluoride is known to disrupt the actin cytoskeleton of protozoa [50] and actin fibers in ameloblasts [51], leading to a disrupted actin cytoskeleton and more rounded cells [51]. Fluoride might thus enhance osteocyte mechanosensitivity by altering osteocyte shape, although the intake would have to be relatively high, considering the dose required to affect the actin cytoskeleton. Second, oleuropein comes to mind. Osteoporosis-related fractures are lowest in Southern Europe, which is likely related to dietary influences [52]. Olive oil and its main compound oleuropein are abundantly present in the Mediterranean diet. The polyphenol oleuropein disrupts microtubules in tumor cells, also resulting in rounded cells and altered cytoskeletal organization [53]. Third, genistein, an isoflavone, and phytoestrogen mostly present in soybeans depolymerizes the microtubules in human A549 epithelium cancer cells and inhibits microtubule polymerization in vitro [54]. One can hypothesize that oleuropein and genistein affect osteocyte shape and primary cilium formation, thereby altering the sensitivity of osteocytes to mechanical loading and affecting bone mass. These findings allow for a hypothetical role for fluoride, oleuropein, and genistein in the mechanoresponse of osteocytes, making them an interesting target for future investigations.

### Estrogen Receptor Alpha

Estrogen is known to have profound bone preserving effects. Decreased estrogen levels following menopause have a strong negative effect on bone mass [55]. On the other hand, administration of exogenous estrogen increases bone mineral density in humans and seems especially beneficial when combined with an exercise regime [56, 57]. Estrogen can affect bone resorption and bone formation after binding to its receptors present on both osteoclasts and osteoblasts, the net effect depending on the type of receptor (i.e., estrogen receptor (ER)α or β) being activated [55, 58]. In addition, estrogen likely alters the response of osteocytes to mechanical loading via the estrogen receptor α and ERβ [59••, 60], thereby affecting bone mass. Mechanical loading is able to preserve osteocyte and osteoblast viability, because ERα, as well as ERβ, activates ERK. Interestingly, the ligand-binding domain of each receptor suffices for mechanosensation, in a ligand-independent fashion, and both plasma membrane localization of the ERα and its interaction with caveolin-1 are required for mechanotransduction [60]. It has been suggested that osteocytes become less sensitive to mechanical stimuli in woman after menopause due to the estrogen loss and alterations in ERα expression, thereby explaining the rapid loss in bone mass associated with menopause [61, 62]. Phytoestrogens are plant-derived compounds with estrogen-like activity, and supplementation with phytoestrogens likely prevents the reduction in BMD associated with menopause and maintains a healthy bone structure [63]. Little is known about the ability of phytoestrogens to modulate ERα expression, but at least genistein enhances ERα expression in MC3T3-E1 mouse osteoblasts [64], suggesting that genistein, similar to endogenous estrogens, is able to modulate ERα expression, and thus potentially alter osteocyte mechanosensitivity.

### Vitamin D Receptor

Not only ERα but also the vitamin D receptor (VDR) is expressed by osteocytes and has been linked to responses of bone cells to mechanical stimuli. The VDR-mediated genomic actions of 1,25 dihydroxy vitamin D_3_ (hereafter referred to as “vitamin D”) occur by coupling of the VDR to VDR response elements (VDRE) in the promoter regions of vitamin D target genes [65]. Genes containing a VDRE often encode for proteins involved in the regulation of osteoclast formation and/or activity, or osteoblast differentiation, such as receptor activator of nuclear factor kappa-β ligand (RANKL) or nitric oxide synthase, the enzyme responsible for nitric oxide (NO) production, e.g., in response to mechanical loading [66]. This prompts the hypothesis that vitamin D enhances the production of signaling molecules by mechanically stimulated osteocytes via genomic actions of the VDR. However, mechanical loading of osteoblasts in vitro through a controlled pulsating fluid flow (PFF) rapidly increases NO production in osteoblasts, but this PFF effect is abolished, rather than enhanced, after 24 h of vitamin D preincubation [67]. Vitamin D may affect mechanical loading-induced NO production independent of genomic VDR action, since it diminishes PFF-induced NO production in osteoblasts derived from genetically altered mice that lack the ability for genomic VDR action [67]. Vitamin D and mechanical loading thus interact at the level of mechanotransduction, at least in vitro.

The role of vitamin D in bone mineral homeostasis is to promote intestinal absorption of calcium and phosphate [68]. Vitamin D regulates calcium and phosphate homeostasis through cross-regulation of PTH, but also via osteoclastogenesis and osteoblastogenesis, calcium and phosphate acquisition, and regulation of anabolic and catabolic gene expression to achieve a proper mineral and skeletal metabolism [69]. Vitamin D deficiency results in increased PTH levels, and PTH reduces the mechanical stress-induced NO production in human primary bone cells in vitro*,* suggesting that vitamin D might affect the response of osteocytes to mechanical loading via PTH [70]. Taken together, vitamin D and mechanical loading are likely to interact, either via the VDR or via modulation of PTH levels.

### Lacunocanalicular Network Architecture and Bone Matrix

PTH may directly affect the *response* of bone cells to mechanical stimulation, but it may also affect the *perception* of mechanical stimuli in vivo. Continuously elevated levels of PTH cause osteocytic osteolysis [71], thereby affecting the architecture of the lacunacanalicular system that forms the niche for the osteocytes embedded within the bone matrix. Altered lacunar shape alters the quantity of local strains occurring around the osteocytes during daily activities and exercise, as outlined elsewhere in this issue, thereby effectively altering the height of the mechanical stimulus experienced by osteocytes [72]. Thus, dietary components that alter osteocyte lacunar shape will most likely interact with mechanotransduction by osteocytes. Regarding matrix strains transduced to osteocytes, dietary components that significantly alter the mineralization of the bone matrix, thereby rendering it more stiff or compliant, will theoretically affect the amount of strain elicited on osteocytes. Dietary components that significantly alter the mineralization of the bone matrix could also affect the ability of osteocytes to maintain an unmineralized layer of matrix surrounding their cell fingers. It is generally assumed that this layer is essential for the transmission of mechanical signals, since loading-induced shifts in interstitial fluid through the canaliculi wherein the osteocyte cell extensions reside is considered an important amplification mechanism for mechanical signals exerted on bone [17]. Factors produced by osteoblasts and osteocytes that affect mineralization include osteocalcin (OC) and matrix GLA protein (MGP). MGP has been found in bone, dentine, cartilage, and soft tissue, including blood vessels, and is associated with the organic matrix and mobilization of calcium. Animal studies show that MGP prevents the calcification of soft tissue and cartilage while facilitating normal bone growth and development. The synthesis of OC and MGP is regulated by calcitriol, retinoic acid, vitamin K, and vitamin D [73]. In addition, it was shown that feeding rats olive extract at 250 mg per day for 12 months increases serum osteocalcin [74]. Whether this also affects matrix mineralization and transmission of mechanical loads towards the osteocytes is unknown. Matrix mineralization is definitely affected by retinol intake in mice, at least under extreme conditions of disturbed matrix mineralization [75]. X-linked hypophosphatemic rickets is caused by inactivation of the PHEX gene. Phex is expressed by very late differentiated osteoblasts and early osteocytes in mice and humans, while it is exclusively expressed by osteocytes in chickens [16]. Feeding mice with a mutant PHEX gene, a retinol-free diet results in a partial rescue of growth plate and bone mineralization defects, while the amount of non-mineralized bone matrix is reduced more than 70%, showing the impact of retinol on the cell-autonomous mineralization defect of Phex-deficient osteoblasts [75]. Whether retinol in the diet will have a similar effect on matrix mineralization in otherwise healthy humans remains to be seen. Taken together, vitamin D may affect the transmission of mechanical signals to osteocytes if it affects lacuna shape, but whether any other dietary component will affect transmission of mechanical signals is uncertain.

### Signaling Molecule Production

Osteocytes produce soluble factors such as nitric oxide (NO), prostaglandin E_2_ (PGE_2_), bone morphogenic proteins (BMPs), Wnts, sclerostin, RANKL, and osteoprotegerin (OPG) in response to changes in mechanical loading. If a dietary component can alter the production of these signaling molecules by osteocytes in response to mechanical loading, it has the potential to enhance bone mass in concert with exercise.

NO is a well-known early mediator of bone formation and is essential for the anabolic response of bone to mechanical loading in vivo [76]. NO also is an essential mediator for the reduced stimulation of osteoclast formation by mechanically loaded osteocytes compared to unloaded osteocytes [77]. Both animal studies and studies in humans support the use of NO donors to prevent bone loss [78]. Since NO is formed during the conversion of l-arginine by NOS, it is feasible that the amino acid arginine plays a role in the process of adaptive bone formation. Several in vitro studies have shown that arginine administration significantly increases NO production, as well as alkaline phosphatase and insulin-like growth factor-I production, and type I collagen synthesis by human osteoblasts and in osteoblasts derived from calvariae of newborn rats [79]. Low concentrations of oleuropein (10^−4^–10^−6^ M) increase NO production via induction of inducible NOS by macrophages in a mouse infection model, thus increasing functional activity of these cells [80]. Both these dietary components could thus have a beneficial effect on NO production by mechanically stimulated osteocytes.

Prostaglandins are generated by the release of arachidonic acid from phospholipids in the cell membrane, followed by conversion of arachidonic acid into prostaglandin G2 and subsequently prostaglandin H2 by COX [81]. Prostaglandin H2 is further isomerized to the biological active prostanoids, such as PGE_2_ [81]. Oleuropein at 10–100 μg/ml reduces the levels of COX-2 seen in inflammation [82]. Another polyphenolic compound, chlorogenic acid, decreases inflammation-induced production of PGE_2_ as well as the expression of COX-2 in RAW 264.7 macrophages [83], indicating a role for polyphenols in inflammation-induced bone resorption. Finally, strontium is known for its effect on the proliferation of pre-osteoblasts and the stimulation of bone formation [84]. In both MC3T3-E1 osteoblasts and MLO-Y4 mouse osteocyte-like cells, strontium stimulates the production of PGE_2_ [85]. Whether oleuropein or chlorogenic acid increases the response of osteocytes to mechanical loading is unknown. Strontium does not seem to have a synergistic effect on the production of PGE_2_ by mechanically loaded osteocytes in vitro, but it does have an additive effect [85].

RANKL is a membrane-bound cytokine and binds to its receptor RANK on osteoclast precursors [86•]. Osteocyte RANKL is a critical mediator of bone loss in response to calcium deficiency [86•]. The severe osteopetrotic (abnormal high bone density) phenotype observed in adult mice specifically lacking osteocyte-derived RANKL indicates that osteocytes are the major source of RANKL during bone remodeling in vivo thereby determining bone mass [87]. Binding of osteoprotegerin (OPG) to RANK results in suppression of osteoclast formation in vivo and in vitro [88]. The ratio OPG/RANKL is therefore considered crucial in osteoclast formation [89]. RANKL is also expressed by osteocytes during bone microdamage [90], and apoptotic osteocytes initiate bone resorption by recruitment of osteoclast precursor cells to the local damage site via RANKL production [91••]. Mechanical loading enhances OPG production and reduces RANKL production by osteocytes. This effect of mechanical loading on RANKL production may be modified via the intake of components that are known to affect RANKL production such as vitamin K. Vitamin K is present as phylloquinone (vitamin K1) and menaquinones (vitamin K2). Examples of the latter are MK-7, MK-8, and MK-9 [92]. Vitamin K2 inhibits osteoclast formation by decreasing RANKL [93]. MK-7 suppresses proliferation, but enhances OPG, RANKL, and RANK gene and protein expression in MC3T3-E1 osteoblasts [94, 95]. This opens the possibility that vitamin K affects the mechanical loading-mediated communication between osteoblasts and osteoclasts. There are many other signaling molecules produced by osteocytes in response to mechanical (un)loading of bone, many of which can likely be affected by dietary components in one way or the other. In this review, we will restrict the discussion to aforementioned signaling molecules as they have been the most extensively studied.

## Conclusions

The increasing number of patients suffering from osteoporosis is accompanied by high costs and decreased quality of life, and therefore new curative and therapeutic approaches are urgently needed. Given the crucial importance of dietary components and physical activity for bone health in general, much is likely to be gained from a regime of daily exercise and a balanced diet. However, more research is needed to obtain a better understanding of the ability of osteocytes to respond to mechanical stimuli in patients with osteoporosis as this might be altered compared to others without osteoporosis [96]. More research is also urgently required to gain a better understanding of the molecular mechanisms involved in the process of mechanotransduction in osteocytes to aid the development of optimal bone-anabolic treatments. In this review, several mechanisms have been identified by which dietary components potentially modulate the beneficial effect of mechanical stimuli on bone health. This review also identified a number of dietary components, e.g., fluoride, oleuropein, (phyto)estrogens, lactoferrin, strontium, vitamin K, and vitamin D with potential for enhancing bone health when applied in combination with mechanical stimuli (Table 1). This list can likely be expanded in the future as research progresses, holding great promise for finding a balanced curative or therapeutic approach for the prevention of age-related bone loss.

**Table 1:** Summary of dietary components potentially able to affect osteocyte properties

	Arginine	Calcitriol	Lactoferrin	Daidzein	Fluoride	Genistein	Strontium	Oleuropein	Retinoic acid	Vitamin D	Vitamin K
Lacuna shape										+/−	
Matrix mineralization		+							+	+	+
Cytoskeletal changes					+	+		+			
ERα expression				+		+					
VDR signaling										+	
Osteogenic diff.			+	+	+	+	+			+	+
Signal molecules	+						+	+		+	+
